# A Bibliometric and Visual Analysis of Exercise Intervention Publications for Alzheimer’s Disease (1998–2021)

**DOI:** 10.3390/jcm11195903

**Published:** 2022-10-06

**Authors:** Xiao-Wei Feng, Maryam Hadizadeh, Lin-Hong Zheng, Wei-Han Li

**Affiliations:** Centre for Sport and Exercise Sciences, Universiti Malaya, Kuala Lumpur 50603, Malaysia

**Keywords:** Alzheimer’s disease, bibliometrics, dementia, exercise intervention, Web of Science, thematic evolution, geriatric disease

## Abstract

Alzheimer’s disease (AD) is the most common cause of dementia worldwide, posing a considerable economic burden to patients and society as a whole. Exercise has been confirmed as a non-drug intervention method in the related literature on AD. However, at present, there are still few bibliometric studies on AD exercise research. In order to fill the gap, this paper aims to intuitively analyze the growth in AD exercise literature published from 1998 to 2021 using bibliometrics, providing historical insights for scientific research circles. The main source of literature retrieval is the Web of Science database. Using the Boolean operator tools “OR” and “AND” combined with keywords related to “exercise” and “Alzheimer’s disease”, we conducted a title search and obtained 247 documents. Using Microsoft Excel, Datawrapper, and Biblioshiny, this study carried out a bibliometric analysis of countries, institutions, categories, journals, documents, authors, and keyword plus terms. The study found that the number of papers published from 2016 to 2021 had the greatest increase, which may have been influenced by the *Global Dementia Report 2015* and COVID-19. Interdisciplinary cooperation and the research results published in high-scoring journals actively promoted research and development in the AD exercise field. The United States and the University of Minnesota system play a central role in this field. In future, it will be necessary to explore the effectiveness and feasibility of multi-mode interventions on an active lifestyle, including exercise, in different groups and environments worldwide. This study may provide a direction and path for future research by showing the global overview, theme evolution, and future trends of research results in the AD exercise field.

## 1. Introduction

According to the “World Population Prospects: 2019 Revision”, one-tenth of the global population will be over 65 years old in 2019, reaching one-sixth of the population in 2050 [1]. It can be predicted that population aging will become a common phenomenon worldwide. Unfortunately, however, many people reaching old age will be threatened by health problems. Research shows that approximately 50 million people worldwide suffer from dementia, most of them older adults. From 2040 to 2050, the number of people suffering from this disease, which leads to memory and cognitive deterioration, is expected to reach 100 million to 130 million [2,3]. Dementia is becoming one of the major challenges faced by all health care systems and society as a whole, presenting a considerable burden to families and caregivers.

The most common form of senile dementia is Alzheimer’s disease (AD), which is a progressive neurodegenerative disease characterized by changes in the brain, such as the accumulation of β-amyloid fragments and the abnormal intracellular accumulation of tau, as well as progressive neuronal loss and cerebral atrophy [4,5,6], leading to memory impairment and cognitive decline, and gradually affecting an individual’s behavior, speech, visual space direction, and motor system [7]. Although extensive basic and clinical research is being carried out at present, no specific drug has been developed for treating this disease [8]. Therefore, appropriate intervention measures are urgently needed to minimize the risk of AD and to help patients control the symptoms.

In 2020, the *Lancet* Commission on Dementia Prevention, Intervention, and Care published the latest life-course model of dementia prevention along with 12 risk factors, which included a lack of physical activity [9]. However, the existing research shows that cardiovascular and metabolic health can be improved through exercise, thus reducing the occurrence of various diseases (such as diabetes and obesity) [10], and preventing a corresponding decrease in motor function [11]. Exercise may also alleviate the symptoms of depression by increasing the level of brain-derived neurotrophic factor (BDNF) [12]. In addition, exercise may have beneficial effects on cerebral perfusion [12] and FNDC5/ irisin levels [13], which contribute to cognitive function [14,15]. Therefore, we must recognize the importance of exercise as a non-pharmacological, viable, and low-cost treatment strategy for the prevention and management of AD.

Bibliometric analysis, as a quantitative statistical tool for academic literature analysis [16], helps to identify continuing trends and emerging areas in specific fields [17]. Compared with traditional systematic evaluation and meta-analysis, bibliometric analysis can reveal the present situation and the evolution of research topics from a more systematic and intuitive level. In recent years, some researchers have combed and analyzed the literature on AD-related research from the perspective of bibliometrics, for example, the brain energy metabolism disorder mechanism of AD [18], publishing trends in AD research [19,20], drug research with regard to AD [21,22,23], genetic research on AD [24], and so on. Specifically, through analyzing the literature from 2000 to 2020, Y.-H. Du et al. found that brain energy disorder plays a central role in the occurrence and development of AD. How to treat or avoid brain energy metabolism disorders may become one of the main research directions in the prevention of AD in the future [18]. In another article, Dong et al., compared the number and influence of AD publications in China and the world from 1988 to 2017, with the author concluding that the global AD research volume has increased dramatically over the past 30 years. The majority of the studies are from the United States, which not only highlights its important role in AD research, but also shows that health care spending is closely related to the economic strength of a country. As a developing country, China has made great progress in AD research, but its academic level still lags behind that of developed countries [19]. At present, the number of papers on AD exercise intervention is increasing with the passage of time, but bibliometric research in this field is still rare. Therefore, employing the bibliometric method, this paper analyzes the sources, authors, countries, institutions, keywords, and theme evolution trend in the exercise research field of AD, providing a practical reference for future clinical research work.

## 2. Materials and Methods

### 2.1. Data Collection and Search Strategies

As the most comprehensive and inclusive database in the world, the Web of Science (WOS) has collected more than 10,000 authoritative academic journals with great impact in fields such as natural science, engineering, and biomedicine. We chose the Web of Science Core Collection (WOSCC) database as the primary data source, which includes A&HCI, BKCI-SSH, BKCI-S, ESCI, CPCI-SSH, CPCI-S, SCI-Expanded, and SSCI. All the data of this study are from public databases, with no human subjects involved, so it has not been reviewed by the ethics committee [25].

Based on published review experience [26], after many attempts to search using different keyword combinations, our final search strategy was to use the Boolean operator tools “OR” and “AND” combined with keywords related to “exercise” and “Alzheimer’s disease” to limit the title search. In addition, asterisks were used to ensure that all relevant words were included in the search. Tl (title) stands for search title according to the Boolean operation. Using keywords in title searches can reduce false-positive results and keep unrelated articles to a tolerable minimum [27]. The specific strategies were as follows: Tl = (“Alzheimer disease” OR “dementia, Alzheimer type” OR “Alzheimer syndrome” OR “AD” OR Alzheimer OR “Alzheimer’s disease” OR “Alzheimer’s dementia”) AND Tl = (“exercise” OR “physical exercise*” OR “sport*” OR “muscle stretching exercises” OR “resistance training” OR “exercise isometric” OR “exercise movement technique*” OR “exercise therapy*” OR “isometric exercise*” OR “muscle stretching exercise*” OR “strength training” OR “strength training program*” OR “training resistance” OR “weight bearing exercise” OR “weight bearing strengthening program” OR “weight bearing” OR “weight lifting exercise” OR “weight lifting” OR “hydrotherapy” OR “aquatic exercise” OR “water exercise” OR “balance exercise”).

**Figure 1:**
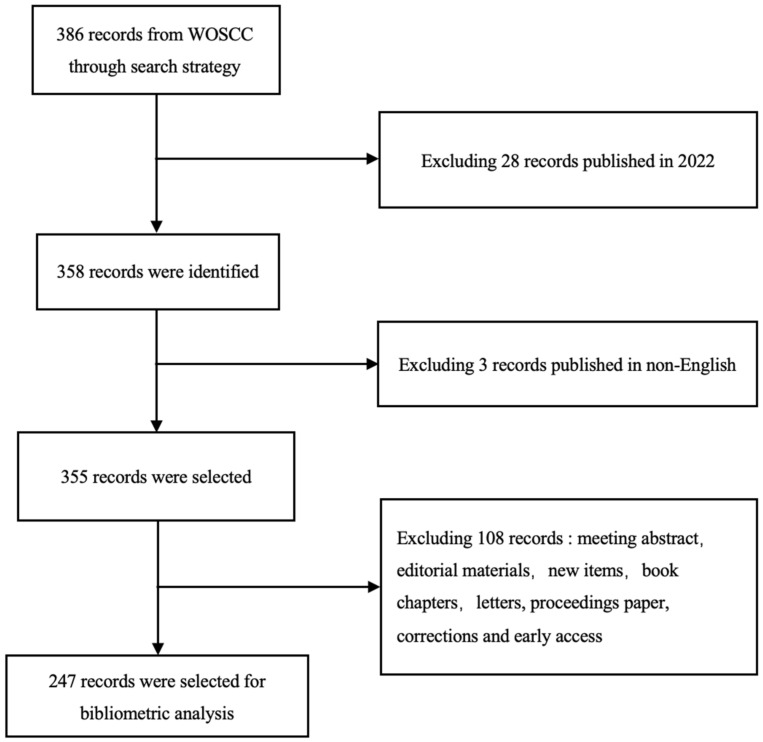
Flow chart of the article selection.

The first document search was conducted on 27 April 2022. In order to avoid the daily deviation of the database, the second search was carried out on 17 July 2022. No start date was selected for both searches. This search strategy found 386 records. We excluded the 28 records published in 2022 as they will continue to be updated. As English, which has a quasi-restrictive effect, was used in the search, non-English articles were excluded. Then, in order to obtain the original findings and reduce the deviation as much as possible, we limited the literature types to original articles and reviews, finally obtaining 247 literature records for in-depth research and analysis (Figure 1).

### 2.2. Bibliometric Analysis

We adopted the bibliometrics analysis scheme proposed by Cobo et al. [28]. On one hand, we analyzed the productivity and influence of scientific publications; on the other hand, we showed the structure and dynamic model of scientific research through scientific maps.

Microsoft Excel was used for classification and statistical procedures. Descriptive information regarding the literature, such as authors, countries, institutions, journals, etc., was obtained through the WOSCC online analysis results. The journal impact factor (IF), Journal Citation Report (JCR) category, and category quartile (CQ) were obtained through the JCR Science Edition (2021). Datawrapper (https://www.datawrapper.de/tables, accessed on 2 July 2022) was used to display the publication category ranking and the publication distribution world map. We also exported the original data in the WOSCC database to BibTex format, and then analyzed Bradford’s law model, keyword analysis, and theme evolution through Biblioshiny.

## 3. Results

### 3.1. Publication Output Analysis

**Figure 2:**
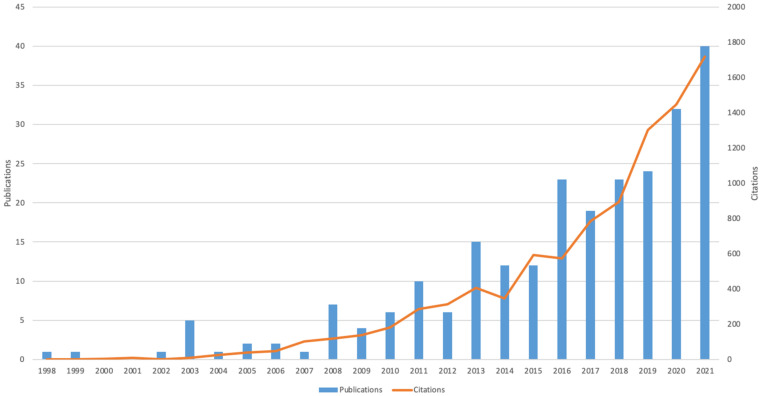
The number of documents and citations per year from 1998 to 2021.

Through searching, we obtained 247 English articles published in the exercise field of AD from 1998 to 2021, with 10,325 citations, of which each article was cited 41.8 times on average, and the H-index was 54. Figure 2 shows that, in recent years, the number of articles and citations in this field has generally been increasing. In 2021 in particular, the number of articles (40) and citations (1725) reached its peak. In addition, it is also worth noting that 161 documents were published during the 2016–2021 period, which is more than half of the total documents (accounting for 65.18%).

**Figure 3:**
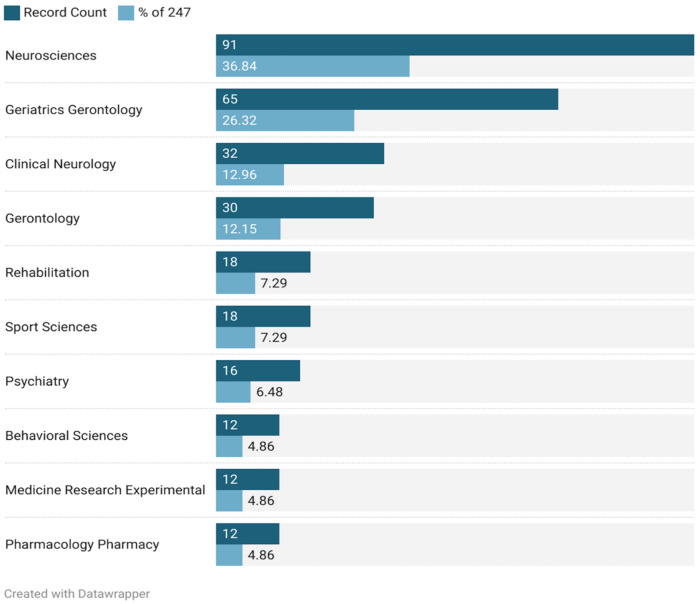
Top 10 categories of exercise research on AD in WOS.

These 247 articles belong to 41 WOS subject categories. We analyzed the top 10 most active categories (Figure 3). The field of neuroscience published the largest number of papers (*n* = 91), followed by geriatrics gerontology (*n* = 65) and clinical neurology (*n* = 32). The publications were mainly focused on neuroscience, gerontology, clinical neurology, rehabilitation medicine, and sports science.

### 3.2. Source Analysis of Periodicals

**Figure 4:**
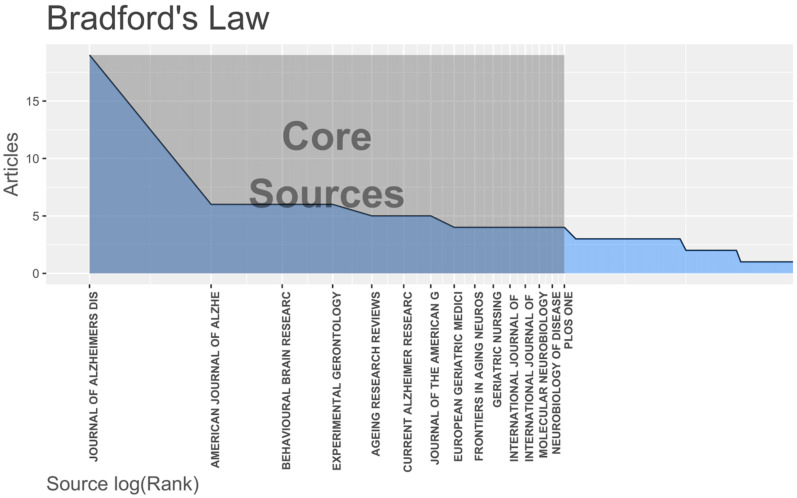
The core journals in the AD exercise research field based on Bradford’s law.

According to our analysis, 139 scientific journals have published exercise research literature on AD. Bradford’s law states that: “If scientific journals are arranged in the order of decreasing output of articles on a specific topic, they can be divided into journal cores more specific to the topic and articles containing the same core” [29]. Therefore, the distribution of scientific output related to a specific topic is extremely unequal [29,30]. Based on this law, the number of core sources with the largest number of exercise publications involving AD is 15 (Figure 4), accounting for 10.8% (15/139) of the whole literature source sample.

We also searched the IF, CQ, and JCR categories of these journals from the JCR Science Edition (2021) (Table 1). With regard to the quartiles, there are six documents in Q1 and Q2, respectively, and three documents in Q3. Among them, the *Journal of Alzheimer’s Disease* published the most articles, with 19 articles, accounting for 7.69% of the total literature (19/247). *Ageing Research Reviews* had the highest IF of 11.788. These journals mostly belong to the neurosciences, geriatrics and gerontology, and clinical neurology categories.

### 3.3. Highly Cited Literature Analysis

We list the 10 most cited studies in Table 2. Eight of these journals appeared in Q1 of the JCR, and include three of the top scoring journals: *JAMA—Journal of the American Medical Association* (IF = 157.355), *Nature Medicine* (IF = 87.241), and *Science* (IF = 63.714). Interestingly, among the 10 most highly cited articles, 6 are in the United States, all of which belong to Q1 journals.

**Figure 5:**
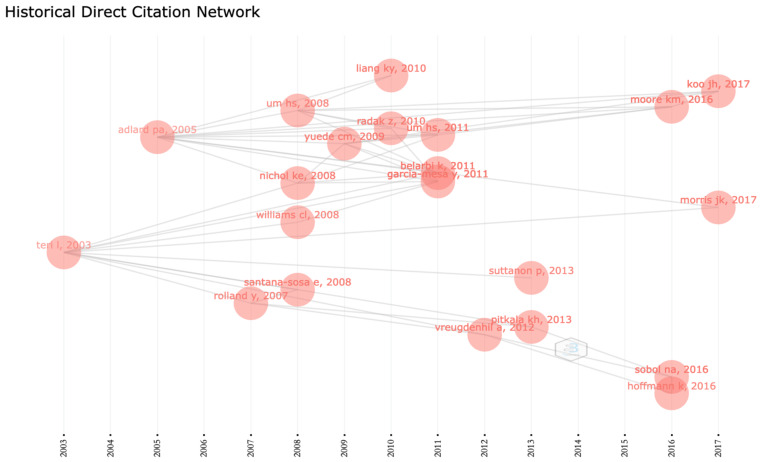
The historical direct citation network of studies on AD exercise research.

Specifically, the top most quoted article published by Adlard et al. in 2005 titled “Voluntary Exercise Decreases Amyloid Load in a Transgenic Model of Alzheimer’s Disease” was of great significance to this topic. As the first study on the effect of exercise on Aβ pathology, the results showed that active exercise could significantly reduce the Aβ load in the TgCRND8 transgenic AD mouse model, proving that exercise, as a simple behavioral strategy, can promote resistance to the development of neuropathology in AD [31]. The second most cited article “Exercise plus behavioral management in patients with Alzheimer disease—A randomized controlled trial” was written by Fountas et al. in 2003. This study showed that sports training combined with caregiver behavior management techniques can improve the physical activity level of Alzheimer’s patients, reduce the incidence of depression, and improve physical health and function. The author emphasized that the potential health benefits of a simple exercise program for Alzheimer’s patients should not be ignored [32]. As can be seen from the historical direct citation network of the AD exercise research literature (Figure 5), these two articles are at the key nodes, respectively, and strongly promote follow-up research from different angles.

### 3.4. Analysis of Main Countries/Regions and Institutions

**Figure 6:**
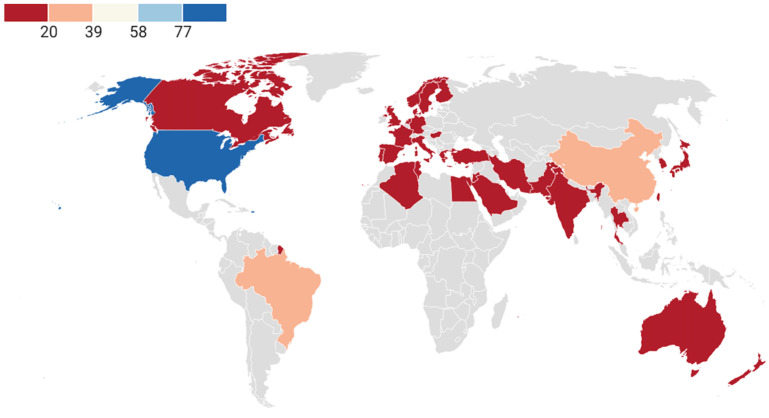
Geographical distribution of exercise research publications in the AD population.

Subsequently, in order to further understand the current situation regarding AD exercise research worldwide, we evaluated different countries and regions. These studies originate from 34 countries/regions. Figure 6 clearly shows the global distribution of AD exercise research publications. The most active countries/regions are mainly in North America, South America, Asia, Europe, and Oceania. In terms of output, the top three countries are the United States (*n* = 96), Brazil (*n* = 33), and China (*n* = 24) (Table 3).

From the perspective of influence, the most relevant countries are the United States (H = 34), followed by Brazil (H = 14), and China (H = 13). The three countries with the highest total citation (TC) are the United States (TC = 5715), Brazil (TC = 964), and South Korea (TC = 610). However, according to the citations per article (CPA), the United States (CPA = 59.53), Australia (CPA = 55.09), and Spain (CPA = 46.42) rank among the top three (Table 3). By analyzing the top institution, apart from Germany’s neurodegenerative disease research center, AD exercise research achievements come from universities all over the world, among which the University of Minnesota system in the United States is the most prominent.

**Figure 7:**
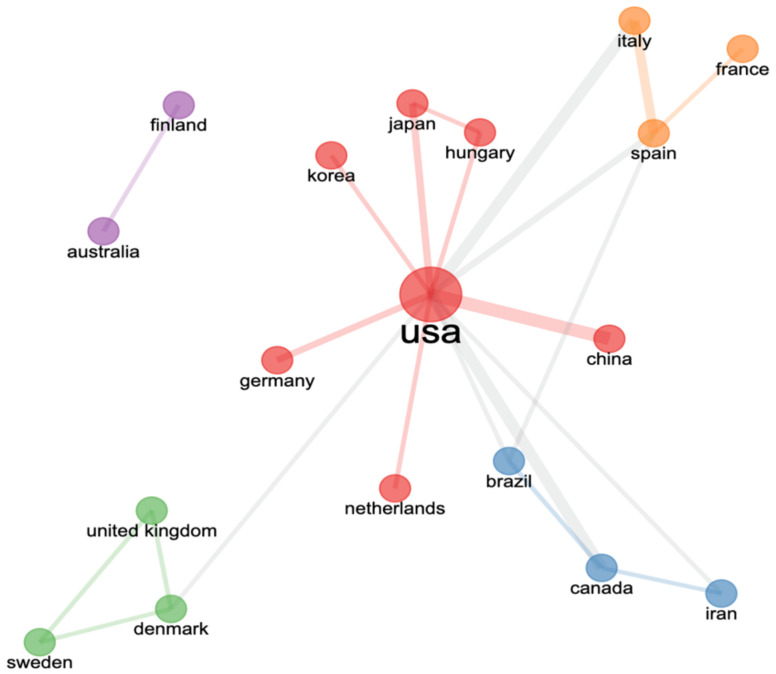
Cooperation between contributing countries on AD exercise research.

Figure 7 shows the cooperation network of countries, with a total of five clusters. Among them, the red cluster shows the strong cooperation between the United States and China, Hungary, Japan, Germany, and Holland. The yellow cluster includes Spain, Italy, and France. The green cluster includes Sweden, Denmark, and the United Kingdom. The blue region includes Brazil, Canada, and Iran. The smallest purple clusters are Australia and Finland. The cooperation among these countries has promoted worldwide exercise research progress on AD.

### 3.5. Analysis of Main Authors

There are 1180 authors who have contributed to AD exercise research in the WOSCC. We list the top 10 most active authors according to the H-index, TC, article, and country (Table 4). In terms of the number of publications, the three most influential authors are Yu, Fang (*n* = 17); Frederiksen, Kristian S (*n* = 11); and Hasselbalch, Steen G (*n* = 11). In terms of impact and TC, the three most important authors are Hasselbalch, Steen G (H = 11, TC = 435); Frederiksen, Kristian S (H = 10, TC = 398); and Waldemar, G (H = 9, TC = 369).

**Figure 8:**
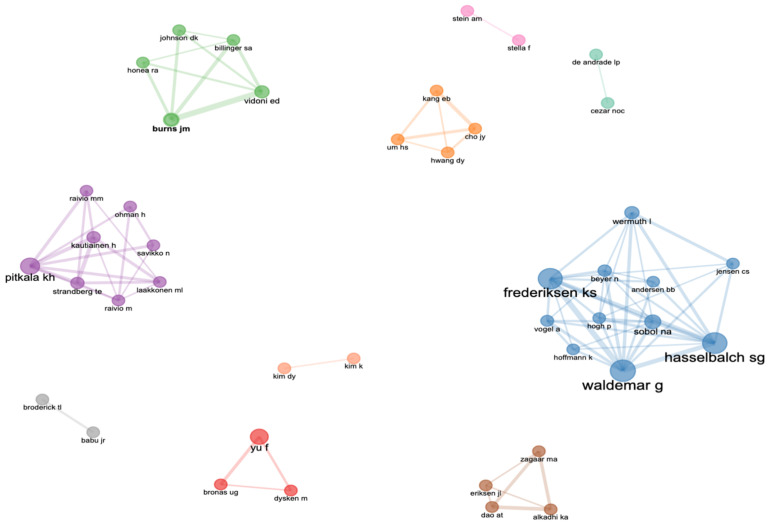
The authors’ collaboration network. (Affiliation—Blue: Denmark; Purple: Finland; Red, Green, Khaki, Gray: USA; Pink, Light Green: Brazil; Orange and Light Orange: Korea).

Interestingly, 80% of the active authors are from Denmark and Finland in Northern Europe, and they exist as a team, respectively. This has also been confirmed in the author’s collaboration network. Figure 8 shows 10 collaboration clusters of authors. The two largest clusters are the blue cluster, representing the Danish research team, and the purple cluster, representing the Finnish research team, both of which have relatively complete cooperation networks.

### 3.6. Thematic Focus and Evolution Trend of the Exercise Field of AD

#### 3.6.1. Keyword Analysis

**Figure 9:**
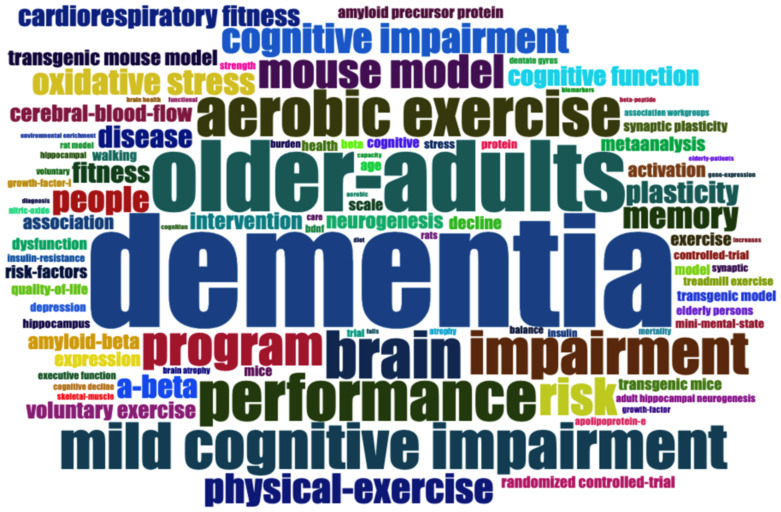
Word cloud visualization of keyword plus terms.

The keywords of publications can help us to quickly determine the theme and concerns of a certain field. We chose keyword plus for keyword analysis because keyword plus can provide a more descriptive trend than the keywords specified by the author [40], and it is conducive to the identification of, and contact with, different research fields [41]. In Figure 9, the word cloud generated by keyword plus shows the commonly used keywords in AD exercise research. The 10 most commonly used words are “physical-activity” (*n* = 90), followed by “dementia” (*n* = 88), “older-adults” (*n* = 55), “brain” (*n* = 37), “aerobic exercise” (*n* = 36), “mild cognitive impairment” (*n* = 36), “impairment” (*n* = 35), “program” (*n* = 30), and “risk” (*n* = 29).

**Figure 10:**
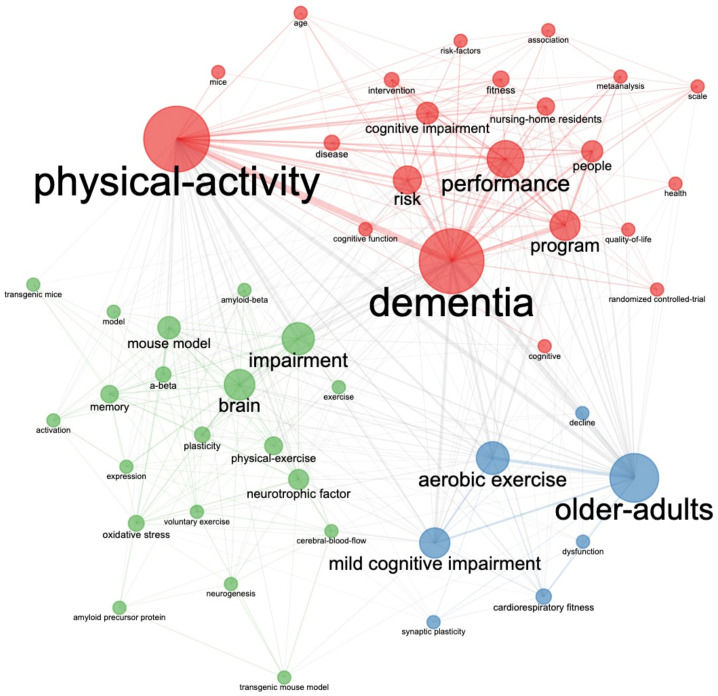
The keyword plus co-occurrence network in AD exercise research.

Next, through keyword plus co-occurrence analysis (Figure 10), we found that the high-frequency keywords in the word cloud all appeared on key nodes, forming three different clusters: red—“physical activity, dementia, and performance”; blue—“elderly, aerobic exercise, and mild cognitive impairment”; and green—“brain, mouse model, and impairment”. This indicates that, in the past 24 years, these three clusters have been the key themes promoting the AD exercise research process.

#### 3.6.2. Theme Evolution

On the basis of knowing the most commonly used keywords, we can further observe the evolution trend of the theme using keyword plus. Theme evolution analysis plays an important role in showing the development of specific research fields, grasping the development direction, and predicting field trends [42]. We reveal the theme evolution in the exercise research field of AD using a Sankey diagram. Different colored nodes represent different research themes, and their sizes are related to the number of keywords included in the topics. The grey flow band between nodes indicates the evolution direction of research topics and the time continuity between them. The wider the band, the more keywords are shared between the two topics, which means a higher relevance.

**Figure 11:**
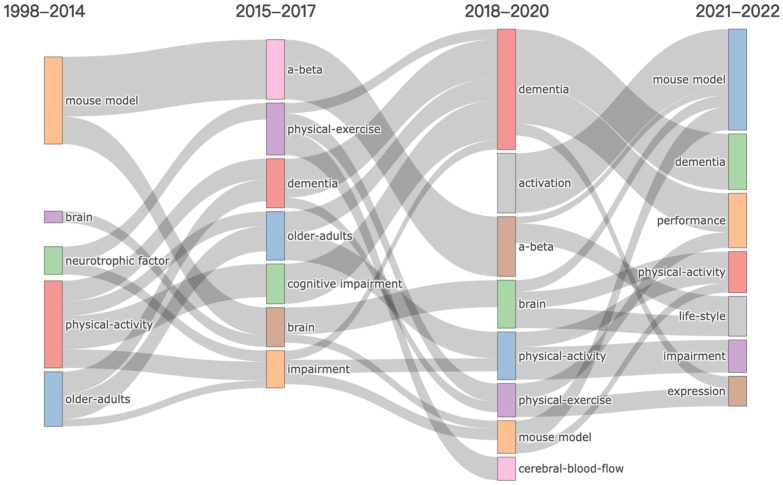
Thematic trend of AD exercise research.

The time span of the theme evolution was constructed from 1998 to 2021 (Figure 11). We used the first 250 keywords in the “Biblioshiny” web software, setting the minimum frequency to 5, as well as the number of representative tags for each topic to 3. At the same time, it was divided into four time periods to be displayed. This setting derives from the author’s subjective judgment and aims to show the best trends in AD exercise research.

In the first stage, from 1998 to 2014, thematic evolution was observed in only five research areas: “neurotrophic factor”, “mouse model”, “brain”, “older-adults”, and “physical-exercise”. This laid the foundation for the later evolution of the theme. In the second stage, from 2015 to 2017, the “older-adults”, “physical-exercise”, and “brain” were still the main themes, with four new thematic areas emerging: “a-beta”, “dementia”, “cognitive impairment”, and “impairment”. This shows that, with the deepening of research, the number of documents is increasing and the theme evolution is ongoing. In the third stage, from 2018 to 2020, two new themes emerged based on the previous stage: “activation” and “cerebral-blood-flow”. In the last stage, 2021–2022, we observed ”performance”, “life-style”, and “expression” appearing.

The evolution of the themes indicates that, since Teri et al. first proposed that exercise and an increased activity level can be a potential treatment for AD in 1998 [43], researchers have begun to pay attention to the beneficial impacts of exercise on cognitive impairment and the daily activity dysfunction of AD patients (e.g., [32,33,35,44,45,46,47,48,49,50]), using mouse models to explore the effects of exercise on brain neural mechanisms (e.g., [13,31,34,39,51,52,53,54,55]). These studies confirm that researchers have been trying to explore the potential protective mechanism of physical exercise on brain aging from mice to humans. Theme evolution analysis can help us understand the present situation and future sustainability of this research field, and can help researchers and related practitioners tap the potential of future research and developments in the AD exercise research field.

## 4. Discussion

This bibliometric analysis of AD exercise research has provided thought-provoking findings. First, since this type of literature first appeared in 1998, it has shown a growing trend. In the last six years (2016–2021) in particular, the number of published studies exceeded half of the total number, which indicates that exercise has gradually received more attention in the AD research field. One possible reason for this is that the Alzheimer’s Disease International Organization’s *Global Dementia Report 2015*, based on a comprehensive update of prior global prevalence, incidence, and cost estimates, emphasized that dementia (including AD and other diseases) will become a severe global public health and social care challenge in the future, and proposed that research results in other fields, including non-drug therapy, should be turned into practical applications in addition to finding a treatment or cure, so as to improve the quality of care and patients’ quality of life [56]. This may prompt more research focusing on the benefits of exercise as a non-drug intervention for patients with different degrees of AD. Subsequently, in 2017, the World Health Organization’s *Global Action Plan for Public Health to Deal with Dementia 2017–2025* was released, which may further promote the development of AD exercise research. Another possible reason is that from 2020 to 2021, COVID-19 swept the world, affecting everyone’s participation in sports, and bringing the sports-related health problems of vulnerable groups to the attention of researchers [57].

The category analysis shows that neurosciences, gerontology, clinical neurology, rehabilitation, and sport sciences are the main categories in the WOS, which indicates that the research on AD exercise is based on a close interdisciplinary cooperation among various traditional scientific categories, using exercise as an intervention strategy to explore the effects on the body, brain, society, and mental health of AD patients [58]. In particular, from the perspective of brain health, although many neurophysiological mechanisms have been proposed in preclinical studies based on animal models as potential ways in which exercise benefits AD, the actual effect of these methods on humans still lacks evidence [50]. For example, exercise may improve the redox state of brain tissue by exerting anti-inflammatory effects, thus improving some physiological characteristics of AD (e.g., increasing the clearance of amyloid plaques and tau protein) [59,60]; and it may have a positive impact on cognitive function and brain structure [61], by promoting the release of BDNF [62,63,64]. Therefore, future research should further clarify the benefits of exercise for AD patients through in-depth interdisciplinary cooperation on the exact biological basis, so as to determine the most effective intervention measures.

Through the analysis of journals and the most highly cited studies, we found that the IF value of the first 15 journals produced using Bradford’s law was generally not high, and only six journals had a value greater than 5. In addition, it is worth noting that there are relatively few papers in this field. Even the journals with the most published papers have only 19 related papers. This shows that, compared with other AD research fields (such as drug development, biomarkers research, etc.), exercise research is still in its primary stage, and more manpower, material resources, and longer research periods are needed to develop and explore in the future. On the other hand, among the top 10 most highly cited studies, eight were from Q1 journals, including *JAMA—Journal of The American Medical Association* (IF = 157.335), *Nature Medicine* (IF = 87.241), and *Science* (IF = 63.714), which are among the best journals in the world. We believe that these high-quality research results with innovative and breakthrough findings affirm the importance of exercise as a non-drug intervention for AD patients, and actively promote research and development in the AD exercise field.

A total of 247 articles from 34 countries/regions were analyzed, with the most active countries/regions in North America, South America, Asia, Europe, and Oceania. Among the top 10 countries and regions, the United States not only has the largest output, H-index, total citation, and average citation in publications, but also owns the world’s leading institution—the University of Minnesota system with a strong national cooperation network. This may be because AD has risen from the 12th most burdensome disease in the United States in 1990 to the 6th in 2016 [65], and its strong economic foundation [19] means that the United States plays an important role in the AD exercise research field. Interestingly, among the top 10 most prolific authors, only two are from the United States, with the remaining eight from Denmark (*n* = 5) and Finland (*n* = 3). The author collaboration network shows that they exist as a team. It may be that Finland and Denmark established national prescription registers earlier in the Nordic countries, which provided excellent opportunities for AD epidemiological research [66], coupled with the high mortality rate of AD in both countries [67], prompting researchers to pay more attention to research in this field.

We have also noticed that, except for Antarctica, where there are no countries or regions, there is still little AD exercise research conducted in Africa. This may be because, according to the *Global Action Plan for Public Health Response to Dementia 2017–2025*, at least 50% of dementia patients in 50 countries will be diagnosed by 2025 [68], while the current diagnosis rate of dementia in Africa is far from this goal [69]. The main reasons for such problems may be a lack of knowledge and understanding, which leads to widespread stigma and discrimination against dementia patients [70], along with an inability to pay for health care services due to the low coverage of health insurance plans [71]. In fact, 75% of Alzheimer’s disease patients worldwide have not been diagnosed, with most of them living in low- and middle-income countries, including African countries [72]. However, dementia has no boundaries or discrimination around the world. It affects people of all sexes, cultures, races, religions, nationalities, sexual orientations, and abilities [73]. Therefore, it is very important to improve the level of prevention, diagnosis, treatment, and care of dementia patients in low- and middle-income countries and regions on the basis of improving the level of awareness and diagnosis, and learning from the experience of intervention measures and services in developed countries. Exercise may be one effective and low-cost intervention.

By analyzing the keyword plus terms commonly used in AD exercise research, it was found that three different clusters formed around the top 10 high-frequency keywords: “physical activity, dementia, and performance”; “elderly, aerobic exercise, and mild cognitive impairment”; and “brain, mouse model, and impairment”. These themes have promoted AD exercise research development. On the other hand, thematic evolution analysis further indicates that researchers have made many advancements in the AD exercise field, ranging from finding that exercise and activity level can be used as potential treatment options for AD, to paying attention to the benefits of exercise on the cognitive deficits and dysfunction in the daily activities of AD patients, as well as exploring the effects of exercise on cerebral neural mechanisms by using mouse models. However, in-depth investigation in animal studies is still needed to find the optimal exercise intervention protocol that can be converted for AD patients. It should be noted that, at present, the key to preventing an increase in dementia patients worldwide is to find a feasible and cost-effective prevention method, which can be applied to people of different backgrounds [74]. Intervention through exercise planning may seem effective, but it is not enough, as existing studies have shown that an integrated approach to reducing the various risk factors for AD may be more successful than a single approach [75]. As a result, the research hypothesis of multi-model intervention should be tested under the guidance of the life-course model of 12 risk factors. To date, Europe has completed three large-scale multi-field prevention trials based on lifestyle: the Finnish Geriatric Intervention Study to Prevent Cognitive Impairment and Disability (FINGER) [76], the French Multidomain Alzheimer Preventive Trial (MAPT) [77], and the Dutch Prevention of Dementia by Intensive Vascular Care (PreDIVA) [78]. However, in order to fully tap the potential and influence of a multi-mode intervention, including exercise, on an active lifestyle, it will be necessary in future to explore its efficacy and feasibility in different groups and backgrounds around the world, and further determine the role of exercise in this multi-mode intervention.

## 5. Conclusions

To the best of our knowledge, this is the first time that a bibliometric study has been conducted in the AD exercise field. This study analyzed the sources, authors, countries, institutions, keywords, and themes of AD exercise research papers published from 1998 to 2021. The results showed that AD exercise research has received extensive attention from researchers in the past six years. However, future research should improve the conversion rate of animal model research results on the basis of in-depth interdisciplinary cooperation, in order to gradually determine the most effective AD exercise intervention measures. The United States and the University of Minnesota system play a key role in this field, but low- and middle-income countries and regions also need to improve their existing AD intervention measures and services on the basis of improving diagnosis awareness and levels. According to the positive results of multi-mode preventive intervention trials in developed countries, it will be necessary to explore the effectiveness and feasibility of multi-mode interventions for the active lifestyles (including exercise) of different people and environments worldwide in the future. In summary, exercise may receive increasing attention in the field of AD, and this study can help researchers to quickly understand the knowledge structure and current hot spots in this field.

## 6. Limitations

There are several limitations to our research. First, the literature was only retrieved from the WOSCC database, which may have led to bias and incomplete inclusion studies. Second, due to the limitations of terms, literature types, time period selection, and languages, our search strategy may not be able to identify all relevant references. Therefore, our research results may be incomplete. Third, in this study, we only used keyword plus to analyze keywords and themes.

## Figures and Tables

**Table 1:** The top 15 most productive journals for AD exercise research (1998–2021).

Journal Name	Articles (%)	IF 2021	CQ 2021	JCR Category
Journal of Alzheimer’s Disease	19 (7.692%)	4.16	Q2	Neurosciences
American Journal of Alzheimer’s Disease and other Dementias	6 (2.429%)	2.632	Q3	Clinical Neurology; Geriatrics & Gerontology
Behavioural Brain Research	6 (2.429%)	3.352	Q2	Behavioral Sciences; Neurosciences
Experimental Gerontology	6 (2.429%)	4.253	Q2	Geriatrics & Gerontology
Ageing Research Reviews	5 (2.024%)	11.788	Q1	Cell Biology; Geriatrics & Gerontology
Current Alzheimer Research	5 (2.024%)	3.04	Q3	Clinical Neurology; Neurosciences
Journal of The American Geriatrics Society	4 (1.619%)	7.538	Q1	Geriatrics & Gerontology; Gerontology
European Geriatric Medicine	4 (1.619%)	3.269	Q3	Geriatrics & Gerontology
Frontiers in Aging Neuroscience	4 (1.619%)	5.702	Q1	Geriatrics & Gerontology; Neurosciences
Geriatric Nursing	4 (1.619%)	2.525	Q2	Geriatrics & Gerontology; Nursing
International Journal of Geriatric Psychiatry	4 (1.619%)	3.85	Q2	Geriatrics & Gerontology; Psychiatry
International Journal of Molecular Sciences	4 (1.619%)	6.208	Q1	Biochemistry & Molecular Biology; Chemistry, Multidisciplinary
Molecular Neurobiology	4 (1.619%)	5.682	Q1	Neurosciences
Neurobiology of Disease	4 (1.619%)	7.046	Q1	Neurosciences
PLoS One	4 (1.619%)	3.752	Q2	Multidisciplinary Sciences

IF = impact factors, CQ = category quartile, JCR = Journal Citation Report.

**Table 2:** Characteristics of the top 10 most highly cited documents on AD exercise research.

TC	Article Title	Journal	Published Year	Country	IF 2021	CQ 2021
559	Voluntary exercise decreases amyloid load in a transgenic model of Alzheimer’s disease [31]	Journal of Neuroscience	2005	USA	6.709	Q1
499	Exercise plus behavioral management in patients with Alzheimer disease—A randomized controlled trial [32]	JAMA—Journal of the American Medical Association	2003	USA	157.335	Q1
423	Exercise program for nursing home residents with Alzheimer’s disease: A 1-year randomized, controlled trial [33]	Journal of the American Geriatrics Society	2007	USA	7.538	Q1
294	Combined adult neurogenesis and BDNF mimic exercise effects on cognition in an Alzheimer’s mouse model [34]	Science	2018	USA	63.714	Q1
273	Exercise-linked FNDC5/irisin rescues synaptic plasticity and memory defects in Alzheimer’s models [13]	Nature Medicine	2019	USA	87.241	Q1
195	Exercise Plays a Preventive Role Against Alzheimer’s Disease [35]	Journal of Alzheimer’s Disease	2010	NETHERLANDS	4.16	Q2
189	Protective Effects of Physical Exercise in Alzheimer’s Disease and Parkinson’s Disease: A Narrative Review [36]	Journal of Clinical Neurology	2013	SOUTH KOREA	2.566	Q3
183	Exercise counteracts declining hippocampal function in aging and Alzheimer’s disease [37]	Neurobiology of Disease	2013	ENGLAND	7.046	Q1
176	Exercise and Alzheimer’s Disease Biomarkers in Cognitively Normal Older Adults [38]	Annals of Neurology	2010	USA	11.274	Q1
174	Effects of voluntary and forced exercise on plaque deposition, hippocampal volume, and behavior in the Tg2576 mouse model of Alzheimer’s disease [39]	Neurobiology of Disease	2009	ENGLAND	7.046	Q1

TC = total citations, IF = impact factors, CQ = category quartile.

**Table 3:** Top 10 countries/regions and institutions with the most research results on AD exercise.

Country	Articles	TC	H-Index	CPA	Top Country Institution *	Top Institution Articles (%)
USA	96	5715	34	59.53	University of Minnesota system	18 (18.750%)
Brazil	33	964	14	29.21	Universidade Estadual Paulista	10 (30.303%)
China	24	558	13	23.25	Nanjing Medical University; Shanghai University of Traditional Chinese Medicine	3 (12.500%)
South Korea	18	610	12	33.89	Korea National Sport University	5 (27.778%)
Canada	13	424	7	32.62	Sunnybrook Health Science Center; Sunnybrook Research Institute; University of Toronto; Western University University of Western Ontario	3 (23.077%)
Denmark	13	459	12	35.31	University of Copenhagen; Rigshospitalet	11 (84.615%)
Finland	12	543	9	45.25	University of Helsinki; University of Oulu	9 (75.00%)
Spain	12	557	8	46.42	European University of Madrid	4 (33.333%)
Australia	11	606	9	55.09	University of Melbourne	4 (36.364%)
Germany	10	394	7	39.4	German Center for Neurodegenerative Diseases (DZNE); Helmholtz Association	3 (30.000%)

TC = total citations, CPA = citations per article, * parallel institutions publish the same number.

**Table 4:** The top 10 authors contributing to AD exercise research.

Author	Country	Articles	H-Index	TC
Yu, Fang	USA	17	9	268
Frederiksen, Kristian S	Denmark	11	10	398
Hasselbalch, Steen G	Denmark	11	11	435
Waldemar, G	Denmark	10	9	369
Hogh, P	Denmark	9	8	325
Kautiainen, Hannu J	Finland	9	8	333
Laakkonen, Marja-Liisa	Finland	9	8	333
Pitkala, Kaisu H	Finland	9	8	333
Beyer, Nina	Denmark	8	7	303
Burns, Jeffrey M	USA	8	6	213

TC = total citations.

## Data Availability

Raw and processed data are available upon request to the corresponding author.
